# Evaluation of UNeXt for Automatic Bone Surface Segmentation on Ultrasound Imaging in Image-Guided Pediatric Surgery

**DOI:** 10.3390/bioengineering12101008

**Published:** 2025-09-23

**Authors:** Jasper M. van der Zee, Aimon M. Rahman, Kevin Klein Gunnewiek, Marijn A. J. Hiep, Matthijs Fitski, Ilker Hacihaliloglu, Ahmed Z. Alsinan, Vishal M. Patel, Annemieke S. Littooij, Alida F. W. van der Steeg

**Affiliations:** 1 Department of Pediatric Surgery, Princess Máxima Center for Pediatric Oncology, 3584 CS Utrecht, The Netherlands; 2 Department of Electrical and Computer Engineering, Johns Hopkins University, Baltimore, MD 21218, USA; 3 Department of Surgical Oncology, Netherlands Cancer Institute, 1066 CX Amsterdam, The Netherlands; 4 Department of Radiology, Department of Medicine, University of British Columbia, Vancouver, BC V6T 1Z4, Canada; 5 Department of Electrical and Computer Engineering, Rutgers University, Piscataway, NJ 08854, USA; 6 Department of Radiology, University Medical Center Utrecht, 3584 EA Utrecht, The Netherlands

**Keywords:** tracked ultrasound, image-guided surgery, pediatric oncology surgery, patient registration

## Abstract

Automatic bone surface segmentation represents an advanced alternative for conventional patient registration methods in surgical navigation technologies. In pediatrics, such technologies require tailored approaches to ensure optimal performance—specifically in patients under the age of ten, whose immature bones have less distinct bone characteristics. In this study, we developed a segmentation model tailored for pediatric patients. We captured 4309 ultrasound images from the bones in the extremities, pelvis and thorax of 16 pediatric patients. The dataset was manually annotated by a technical physician and sample-wise validated by a pediatric radiologist. A UNeXt deep learning model was trained for automatic segmentation. The segmentation performance was evaluated using the mean centerline Dice score and the mean surface distance. A mean centerline Dice score of 0.85 (SD: 0.13) and a mean surface distance of 0.78 mm (SD: 1.15 mm) were achieved. No important differences in performance were observed for patients younger than the age of ten compared to older patients. Our results demonstrate that the segmentation model detects the bone surface with sufficient accuracy, enabling precise and effective patient registration. The model performs sufficiently across different pediatric age groups, making it a viable tool for integration into ultrasound-based patient registration in image-guided pediatric surgery.

## 1. Introduction

Patient registration for image-guided surgery is the intraoperative aligning process of the preoperative 3D planning with the actual patient [1]. In conventional approaches, this process often involves the identification of superficial anatomical landmarks, such as pinpointing the frontonasal junction in neurosurgery, to establish a point-based patient registration. However, for some clinical cases, no superficial bone structures are available and an intraoperative cone beam computed tomography scan is preferred for patient registration. Despite its advantages for accuracy, this procedure comes with several limitations, such as significant workflow interruptions and ionizing radiation exposure, which is especially harmful in pediatrics [2].

In recent years, ultrasound-based patient registration has become a viable alternative owing to its capacity for real-time imaging while remaining free from ionizing radiation [3,4,5,6,7]. Particularly, the combination of ultrasound imaging with a tracking system (i.e., intraoperative tracked ultrasound) serves as a viable imaging modality for the detection of the bone surface for patient registration. The specific acoustic characteristics of the bone’s appearance can be used to develop automatic segmentation models to derive a 3D point cloud of the bone surface [8,9]. In adults, several researchers have developed models for a variety of applications with promising clinical results. In a recent study, tracked ultrasound-based bone registration in 30 adult patients resulted in a median registration accuracy of 1.2 (IQR: 0.9–1.2) mm at the bone surface and 2.6 (IQR: 1.3–5.7) mm at target lymph nodes in adult oncology surgery [10].

Automatic segmentation has emerged as a prominent approach for real-time ultrasound segmentation, forming a vital step in ultrasound-based patient registration [11,12]. Specifically, different types of algorithms have been widely adopted for bone surface segmentation, as highlighted in different systematic reviews [13,14]. In the majority of the included studies, a U-Net architecture was used for automatic segmentation, and in a more limited number of studies, segmentation was performed using either a local phase symmetry or a shadow peak algorithm [8,15,16,17,18]. Particularly, the existing superiority of deep learning approaches in terms of their robustness, accuracy and computation times leads to a preference for real-time detection to be used in ultrasound-based image-guided surgery [14,19]. In the case that surgical navigation is indicated for tumor resection of the ribs, the presented models are not specifically trained for this specific anatomy [20]. Preferably, an automatic segmentation model should be able to detect the bone surface in a broad variety of anatomical locations, such as the bones in the extremities (e.g., femur, tibia, humerus), thorax (e.g., sternum, ribs) and pelvis (e.g., os pubis, os ilium).

Although the results of ultrasound-based patient registration methodologies are promising in adults, bone segmentation presents additional challenges in pediatrics because of marrow maturation processes during growth. Throughout childhood (i.e., ages 1–10 years), red bone marrow gradually converts to yellow marrow from the bone center outward, leading to denser bones. This bone maturation progresses from distal to proximal locations: occurring earlier in the hands/feet than in the pelvis/spine [21,22]. Immature bones cause less distinct cortical lining, which can impair the performance of a model that is trained exclusively on adult imaging data for bone surface detection.

Translating the recent advances in patient registration methods for image-guided surgery from adults to pediatrics, a tailored approach is required for optimal performance. Currently, models trained specifically on pediatric imaging data are lacking. Moreover, as an extra-cranial solid tumor resection can be performed on any anatomical location in pediatric oncology surgery, an automatic segmentation model that is trained on different types of bones is required. In this study, we evaluated a novel deep learning-based UNeXt segmentation model, trained on 2D imaging data of pediatric patients containing a wide variety of bone types [23].

## 2. Materials and Methods

### 2.1. Patient Population and Data Split

**Figure 1:**
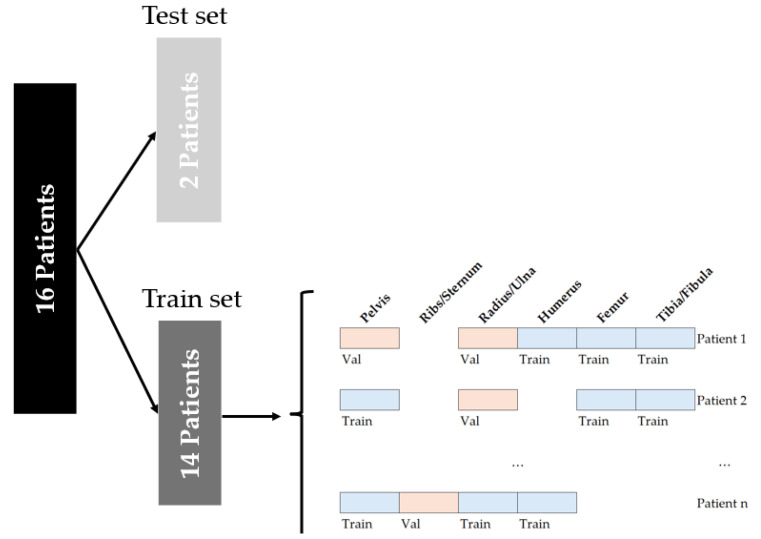
The data split on the imaging data. The imaging data for the training set was divided into a validation and training set, based on an even distribution of age and anatomical locations.

Ultrasound images were acquired from 16 pediatric patients, including 7 patients under the age of 10 years, following our standard intraoperative imaging procedure. Ultrasound images of osseous structures in the extremities, pelvis and thorax were acquired. In total, the dataset consisted of 4672 ultrasound images, along with 363 ultrasound images with empty labels to reduce the false detection rate. Dataset characteristics are detailed in Table 1. The data was distributed among a train, validation and test set in an 80:10:10 split based on an even distribution of anatomical location per dataset, as shown in Figure 1. The test set consisted of data coming from two patients, one above and one below the age of ten. To reduce overfitting, a five-fold cross-validation was performed, resulting in a weighted model.

### 2.2. Data Acquisition and Image Processing

Data acquisition was performed on the Hitachi Aloka (Hitachi Medical, Tokyo, Japan) ultrasound machine using both the linear L441 probe, for the bones in the extremities and thorax, and the C35 curved abdominal probe for the pelvis. During scanning, several instructions were taken into account: the probe was orthogonally positioned in relation to the bone surface, the bone surface was located on different imaging depths (i.e., 2–6 cm) and different ultrasound gains and both axial and transversal slice orientations were used. For bones in the extremities, both the diaphysis as well as the epiphysis with more pronounced anatomical morphologies were included, and for the pelvis, different locations were scanned (e.g., pubic bone, iliac crest lateral and medial). The imaging data was streamed to a computer workstation equipped with an Intel(R) Core(TM) i7-13800H CPU, 32 GB of RAM and a NVIDIA GeForce RTX 4080 GPU. Streaming was established with a frame grabber AV.io HD (Epiphan System Inc, Ottawa, ON, Canada) and a sampling frequency of 4 frames per second. Data was stored as a sequence format in 3D Slicer (version 5.8.1) [24].

**Figure 2:**
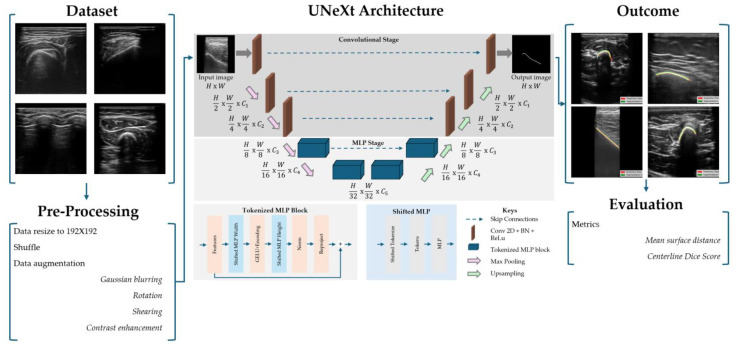
The workflow for model development is illustrated. The workflow was divided into different phases. The UNeXt architecture was built, containing different convolutional and multilayer perception (MLP) stage layers. The outcomes were evaluated using two metrics: mean surface distance and centerline Dice score. The overview of the architecture was adapted from Valanarasu et al. [23]., with author permission.

The dataset was manually labeled by a technical physician who was trained to identify bone surface on ultrasound images by the pediatric radiologist. The images of the training set were sample-wise validated by the pediatric radiologist, while all test images were fully validated. The annotation was performed using the Single Slice Segmentation software package available in the SlicerIGT software plugin in 3D Slicer [25]. This tool enables the operator to manually select points along the bone surface. The toolbox generates a line using a Kochanek spline interpolation method, ensuring a smooth, anatomically consistent representation of the bone surface. Training data was resized (192 × 192), shuffled and augmented to increase heterogeneity among images. Augmentation was performed on an arbitrary quarter of the training set and consisted of Gaussian blurring (σ = 5), rotating (−10° up to 10°), shearing (factor −0.2 up to 0.2) and contrast adjustments (0.25 to 1.75) [26]. A schematic overview of model development is shown in Figure 2.

### 2.3. UNeXt Architecture

Our proposed architecture builds upon a hybrid encoder–decoder framework inspired by U-Net, enhanced with tokenized MLP modules and shift-based MLP blocks for efficient spatial reasoning. The architecture, referred to as UNeXt [23], is designed to capture both the local and global context using a combination of convolutional and token-mixing mechanisms. The UNeXt architecture is schematically visualized in Figure 2.

The encoder consists of three successive convolutional stages, each comprising a convolutional layer followed by batch normalization, ReLU activation and 2 × 2 max-pooling. These stages gradually increase the number of channels from 16 to 128 while reducing spatial dimensions. The feature maps are then tokenized using an overlapping patch embedding module that projects spatial features into a sequence of tokens for MLP-style processing. The bottleneck and deeper encoder stages are constructed using shifted MLP blocks, which replace traditional attention mechanisms with spatially shifted grouped convolutions followed by MLP layers. This allows the model to aggregate the global context at a reduced computational cost. Two such blocks are used sequentially at increasing depths, operating over embedded dimensions of 160 and 256.

The decoder mirrors the encoder via a multi-stage upsampling path with bilinear interpolation and convolutional refinement. Skip connections from the encoder stages are fused via element-wise addition at corresponding resolutions. Decoder stages also incorporate token-based processing, including MLP refinement of upsampled features, enabling strong recovery of spatial details. To ensure accurate mask prediction, we use a final 1 × 1 convolution to reduce the number of channels to 1 for binary segmentation. The total parameters for all layers of this model are 1.38 million, as shown in Table 2.

### 2.4. Model Parameters

A combined loss function was used to account for the class imbalance and spatial overlap. The model was first trained on foreground and background characteristics and was thereafter fine-tuned for the specific bone surface morphology. In the first ten epochs the loss function depended solely on the Weighted Binary Cross-Entropy (WBCE) loss, with a set weight of 20, as a warm-up to handle class imbalances. After ten epochs, a dynamic loss function was computed by increasing the fraction of the two different loss functions to fine-tune the performance. In this dynamic loss function, the WBCE loss was combined with a centerline Dice loss function, which was computed according to the publicly available method of Shit et al. [27]. At the last epoch, the combined loss function is 50:50, which is calculated by the WBCE loss and the centerline Dice loss. The model parameters were optimized using an AdamW optimizer, with a learning rate of 1 × 10^−3^ and a weight decay of 5 × 10^−4^. A ReduceLROnPlateu scheduler was used to reduce the learning rate by 0.5 every 3 consecutive epochs if the validation loss had not improved. The model with the best centerline Dice score on the validation set was saved during training.

### 2.5. Evaluation Metrics

The centerline Dice score was first described by Shit et al. [27] and calculates the intersection of the segmentation masks and their morphological skeletons (1). Based on the segmentation $V_{L}$ and the prediction $V_{P}$, two skeletons ${(S}_{P},S_{L})$ are defined. Following the Topology Precision, $T_{Prec}$, and Topology Sensitivity, $T_{sens}$, the centerline Dice can be computed. To illustrate, $T_{Prec}$ describes the fraction of the predicted skeletons within the segmentation, making this specific measure sensitive to false positives.

The mean surface distance error is calculated by computing the Euclidean distance between all segmented and predicted surface points on the skeletons. This function computes the distance between each pair of points from the segmentation and the prediction (2). The resulting array is then adjusted to identify the closest predicted surface point to each segmentation point. Finally, the mean surface distance is computed as the average of all distances between the segmentation points and their nearest predicted surface points (3).

(1) $$clDice\left(V_{P},V_{L}\right)=2*\frac{T_{Prec}\left(S_{P},V_{L}\right)*T_{sens}\left(S_{L},V_{P}\right)}{T_{Prec}\left(S_{P},V_{L}\right)+T_{sens}\left(S_{L},V_{P}\right)}$$

(2) $$Distance\;array=cdist(S_{P},S_{L})$$

(3) $$Mean\;surface\;distance=\frac{1}{N}\sum_{i}^{N}min\;(Distance\;array)$$

The minimal metric for this model should be a centerline Dice score of 0.75 and a mean distance error less than 2 mm, for efficient bone-based patient registration. In future surgical navigation applications, the predicted bone surface will result in a 3D point cloud that will be matched with the 3D model derived from the surgical 3D planning [7,10,28]. In the subsequent bone registration step, which is comparable to surface-based registration, all predicted bone points that are further away from the bone surface of the 3D planning can now be removed for a more accurate registration. This iterative registration procedure begins with an initial alignment of the patient to the ultrasound probe. If the segmentation algorithm detects surface points that are at a larger distance than a pre-defined threshold (e.g., up to 2 or 5 mm), such points can be filtered out. Therefore, the minimal outcome metrics are set to be relatively tolerant, and the number of false positives may not lead to significant impacts on the registration accuracy.

## 3. Results

**Figure 3:**
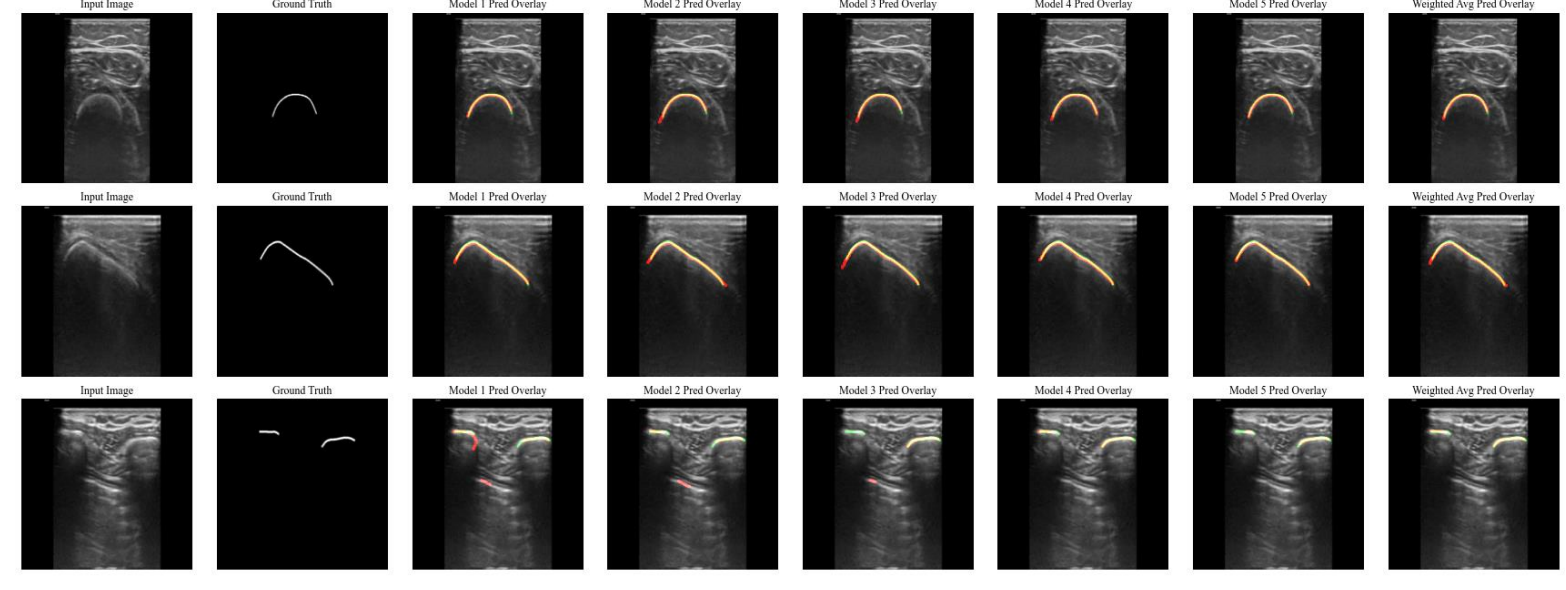
Examples of the predictions per model and the weighted model for images of the femur (**top row**), pelvis (**middle row**) and ribs (**bottom row**). The bone surface is shown in white (label), green (ground truth) and red (prediction).

The five individual models showed similar results, shown in Table 3, with a cumulative weighted centerline Dice score of 0.85 (SD: 0.13) and a mean surface distance of 0.78 mm (SD: 1.15 mm) for the weighted model. The performance per model and the weighted model are visualized in Figure 3, with the resulting predictions on three test images containing bones of the extremities, pelvis and ribs. Specifically, for some images of the ribs, false positives were observed on the pleural surface. Although the individual models performed slightly differently on the same input image, the resulting weighted model corrected such small deviations, as illustrated in the bottom row of Figure 3.

**Figure 4:**
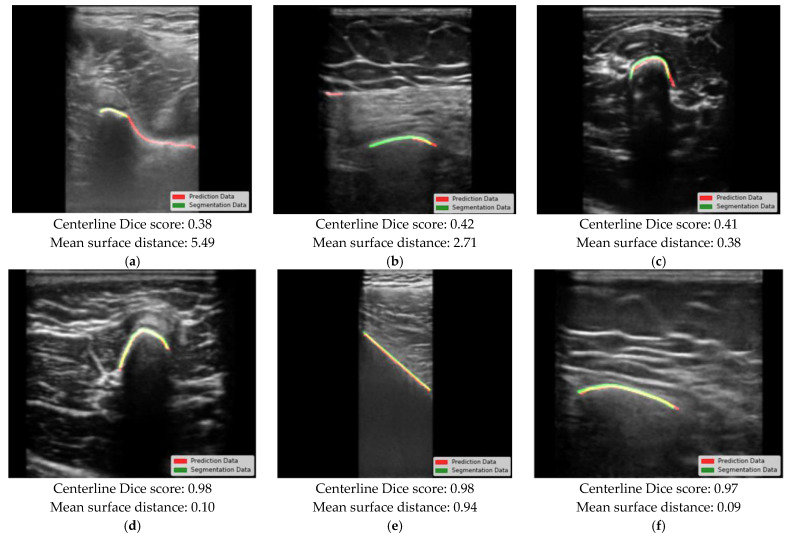
The worst (**a**–**c**) and best (**d**–**f**) performance of model 4. The bone surface is shown in green (ground truth) and red (prediction).

The three worst and three best predictions are demonstrated by model 4, as shown in Figure 4. The tested models showed a minor difference in performance for patients younger than 10 years, but all results were within the clinical requirements. In some cases, an oversegmentation was observed, resulting in a prolonged bone surface in the prediction. False positives were mostly seen at muscle layer boundaries or at the fascia (e.g., between the radius/ulna or tibia/fibula). On average, the bone surface was predicted to be 0.05 mm in the direction of the ultrasound probe. The average computation time per million pixels was 0.96 ms (SD: 0.17 ms).

## 4. Discussion

In this study, an automatic bone segmentation model tailored to pediatric ultrasound images was developed and evaluated. The UNeXt model showed an acceptable performance, with an overall mean surface distance of 0.78 mm (SD: 1.15 mm). Based on the results, we consider the model suitable enough for pediatric patient registration in surgical navigation systems for different anatomical locations.

The presented performance evaluation of the segmentation of the bone surface on ultrasound images is challenging [13,14]. Although metrics such as the Hausdorff distance and the Sørensen–Dice score are commonly used to evaluate performance, these metrics are impractical for the evaluation of the segmentation of single lines [29]. The Hausdorff distance will inevitably result in a measurement between the end and starting points of the segmented lines, which will not reflect the correct distance error. Similarly, the Sørensen–Dice score may yield a low value even when the alignment of the segmented line is acceptable. Therefore, alternative evaluation metrics had to be considered to evaluate the correspondence between the two surface lines. As outlined in the scoping review of Pandey et al. [14], both distance and overlap metrics should be used, which in this study represent the mean surface distance error and the centerline Dice score, respectively.

Oversegmentation was observed (i.e., a detected prolonged bone surface), and this may suggest that the annotated bone surface in the image label was too short in some cases. This can be caused by subtle intensity differences that were not addressed during the annotation nor the dataset validation. Nevertheless, these undersegmentations do not strongly affect the automatic registration for which this algorithm is designed. Furthermore, the detection of false positives may not influence the subsequent registration either. However, postprocessing steps may be needed, such as the removal of distant false positives (e.g., a point at the fascia at 5 mm from the bone) or filtering methods based on probability [10]. This may lead to the integration of advanced methods to detect the incidence angle of the ultrasound wave and may classify true bone points in case they were detected while scanning the bone orthogonally [17].

The performance of our model is comparable to the pediatric models described by Hers et al. [30] and El-Hariri et al. [19], which are unique in the presence of the pediatric imaging data in the training set. Their models, trained on an imaging dataset containing ultrasound images of the femoral head and pelvis from newborn patients, are able to automatically detect the femoral head with a Dice score around 0.80, which is comparable to our weighted model trained on different bone structures. Moreover, comparing our model to models trained on adult data, our performance is in line with the scores reported in the literature. In the review paper by Holhman et al. [13], which reviewed 58 papers with models trained on adult imaging data, 17 out of 30 papers reported Dices scores above 0.80. Moreover, the mean surface distance was described in only 13 papers, and in 9 papers the score was below 0.60 mm.

Moreover, although small differences were observed in the segmentation performance between images of patients below and above the age of ten, the clinical requirements were met. The poorer performance in the younger age group, of whom 5/9 were three years old or younger, was indeed most likely caused by immature bones, underlining the specific need for this tailored approach. Differently from pervious approaches, we used a UNeXt architecture instead of the commonly used U-Net architecture [23]. This model is a new deep learning architecture known for its high inference speed and low computational complexity. Resultingly, the prediction time per million pixels is potentially one of the fastest compared to the computation times reported in the review paper by Holhman et al. [13], making this model suitable for real-time predictions at high framerates.

Different bone structures have different ultrasound appearances and thus potentially different model performances. Specifically, the ribs are an unique bone structure that is more challenging to segment than, for instance, a femur. The ribs are known as different superficial curvilinear structures on ultrasound, with a highly reflecting pleural surface in between, potentially resulting in a large ratio of false positives, as shown in the bottom row of Figure 3 [31]. Although the segmentation of the ribs is important for surgical navigation in this area, automatic rib segmentation has not been extensively described previously. In our study, we tried to obtain an one-size-fits-all model that is robust and applicable for bone-based patient registration in all anatomical areas.

To the knowledge of the authors, this study presents the first results for automatic bone segmentation, with a broad variety of bone structures, to be used in ultrasound-based patient registrations tailored to pediatric patients. A limitation of this study is the relatively small sample size for different anatomical locations and patients numbers, which is inevitable for machine learning in pediatric cohorts [32]. Future studies should include a larger amount of imaging data, captured with different imaging devices, to obtain an even more generalizable and robust model. To improve the model performance by adding more samples in the training set and without scanning more children, available adult image samples may be added to the training set, such as the publicly available UltraBones100k dataset of Wu et al. [33], and more pediatric imaging data can be added to the validation and test set. Moreover, as we captured all ultrasound images with the same ultrasound device, the resulting model may not be directly generalizable for other imaging devices. Nevertheless, the results of this study are sufficient to integrate the presented model in a surgical navigation technique, which is comparable to the setup described by Hiep et al. [10] and Van der Zee et al. [34].

## 5. Conclusions

We present a UNeXt model that is able to accurately locate the bone surface within a 1 mm distance and 1 ms. The model is sufficient and fast, making it suitable for automatic bone surface segmentation for all pediatric ages and bone structures. In the future, extended efforts will be undertaken to make the model more robust and applicable for patient registration in surgical navigation for pediatric oncology.

## Figures and Tables

**Table 1:** An overview of the number of images per anatomical structure per age group. The younger group (<10 years, *n* = 7) consisted of patients aged 1 (*n* = 3), 2, 3, 6 and 9 years. The older group (≥10 years, *n* = 9) included patients aged 12, 13, 14 (*n* = 4), 15, 17 and 19 years.

Osseous Structure	0–9 Years (*n* = 7)	10–19 Years (*n* = 9)	Total (*n* = 16)
	Mean Age: 3.5 +/− 2.7 Years	Mean Age: 14.7 +/− 2.0 Years	Mean Age: 9.8 +/− 6.0 Years
Pelvis	699	491	1190
Ribs/Sternum	420	277	697
Radius/Ulna	154	126	280
Humerus	365	378	743
Femur	176	386	562
Tibia/Fibula	442	395	837
**Total**	**2256**	**2053**	**4309 + 363 empty labels = 4672**

**Table 2:** The number of parameters for each layer of the UNeXt architecture.

Layer	Type	Number of Parameters
Encoder1_Conv2D	Conv2d	160
Encoder1_BatchNorm	BatchNorm2d	32
Encoder2_Conv2D	Conv2d	4640
Encoder2_BatchNorm	BatchNorm2d	64
Encoder3_Conv2D	Conv2d	36,992
Encoder3_BatchNorm	BatchNorm2d	256
PatchEmbed3_Conv2D	Conv2d	184,480
PatchEmbed4_Conv2D	Conv2d	368,896
ShiftMLP1_fc1	Linear	25,760
ShiftMLP1_fc2	Linear	25,760
ShiftMLP2_fc1	Linear	65,792
ShiftMLP2_fc2	Linear	65,792
Decoder1_Conv2D	Conv2d	368,800
Decoder2_Conv2D	Conv2d	184,448
Decoder3_Conv2D	Conv2d	36,896
Decoder4_Conv2D	Conv2d	4624
Decoder5_Conv2D	Conv2d	2320
Final_1 × 1_Conv	Conv2d (1 × 1)	17
		Total: 1,375,712 parameters

**Table 3:** Segmentation performance after five-fold cross-validation on test set. The direction of the segmentation indicates that the prediction was performed in the direction towards or away from the ultrasound probe for positive or negative distances, respectively.

Model	Centerline Dice Score (Mean ± SD)			Mean Surface Distance (Mean ± SD), Direction of Segmentation [mm]			Computation Time Per Million Pixels
	All Ages	<10 Years	>10 Years	All Ages [mm]	<10 Years [mm]	>10 Years [mm]	All Ages [ms]
1	0.80 ± 0.15	0.77 ± 0.17	0.83 ± 0.12	0.86 ± 1.04, 0.00	0.93 ± 1.35, −0.17	0.77 ± 0.69, +0.13	1.21
2	0.81 ± 0.14	0.78 ± 0.17	0.84 ± 0.10	0.82 ± 1.00, +0.07	0.98 ± 1.37, −0.07	0.69 ± 0.52, +0.18	0.79
3	0.82 ± 0.14	0.79 ± 0.17	0.84 ± 0.11	0.91 ± 1.08, +0.11	1.09 ± 1.46, +0.05	0.77 ± 0.61, +0.16	0.84
4	0.84 ± 0.14	0.81 ± 0.17	0.86 ± 0.10	0.48 ± 1.30, +0.21	0.74 ± 1.89, +0.19	0.28 ± 0.33, +0.21	1.12
5	0.84 ± 0.14	0.83 ± 0.16	0.85 ± 0.11	0.51 ± 0.93, +0.07	0.65 ± 1.23, +0.09	0.40 ± 0.48, +0.05	0.85
Weighted model	0.85 ± 0.13	0.82 ± 0.16	0.87 ± 0.09	0.78 ± 1.15, +0.05	1.0 ± 1.63, +0.07	0.61 ± 0.46, +0.03	Not specified

## Data Availability

The data and segmentations used in this study are not publicly available due to patient confidentiality reasons. The trained model is available from the corresponding author upon reasonable request.

## Abbreviations

The following abbreviations are used in this manuscript:

3D	Three-dimensional
IQR	Inter quartile range
SD	Standard deviation
